# Death from Liver Failure despite Lamivudine Prophylaxis during R-CHOP Chemotherapy due to Rapid Emergence M204 Mutations

**DOI:** 10.1155/2013/454897

**Published:** 2013-07-29

**Authors:** Lay Lay Win, Jeff Powis, Hemant Shah, Jordan J. Feld, David K. Wong

**Affiliations:** ^1^Hepatology, Toronto Western Hospital, University of Toronto, 399 Bathurst Street, 6b-176, Toronto, ON, Canada M5T 2S8; ^2^Infectious Diseases, Toronto East General Hospital, 825 Coxwell Avenue, East York, ON, Canada M4C 3E7

## Abstract

*Background*. Rapid and early emergence of clinically significant LAM resistance is thought to be unlikely during the first year of treatment, and as a result LAM is thought to be a reasonable choice as a first line agent for prophylaxis during chemotherapy. *Aim*. To report fatal HBV reactivation despite appropriate LAM prophylaxis in two previously treatment-naive individuals undergoing R-CHOP chemotherapy. *Case Presentation*. Case 1 is a 65-year-old man with chronic HBV infection: HBeAg-negative, HBV DNA 6.65E5 IU/mL, ALT 43 IU/L, and Fibroscan 4.4 kPa, consistent with F0, who was diagnosed with lymphoma that was treated with R-CHOP and LAM prophylaxis. HBV DNA fell to 2.18E1 IU/mL within 2 months of starting LAM. Four months after chemotherapy, despite ongoing LAM of 7-month duration with confirmed adherence, severe asymptomatic hepatitis was noted during routine monitoring with ALT 1019 IU/L, HBeAg negative, HBV DNA 1.43E7 IU/mL, and genotyping confirmed L80I and M204I mutations. He died 14 days after flare diagnosis despite a switch to tenofovir (HBV DNA had fallen to 1.94E5 IU/mL 2 weeks after starting tenofovir). Case 2 is a 50-year-old man who was found to have HBeAg-negative hepatitis B, ALT 37 IU/L, and no clinical features of cirrhosis (platelets 283, APRI 0.19) after lymphoma diagnosis. Lymphoma was treated with R-CHOP and LAM prophylaxis. Pretreatment HBV DNA was not done but was 8.90E4 IU/mL 3 weeks after starting LAM and 3.96E3 IU/mL 3 months after starting LAM. Two months after chemotherapy, despite ongoing LAM of 7-month duration with confirmed adherence, severe symptomatic hepatitis presenting with jaundice, abdominal pain, and confusion was noted. ALT 902 IU/L, HBeAg negative, HBV DNA 1.02E8 IU/mL, and genotyping confirmed L80I, M80V, and M204V/S mutations. He died 3 days after flare diagnosis despite the addition of tenofovir. *Conclusion*. Lamivudine should not be used for prophylaxis of patients with chronic hepatitis B with detectable HBV DNA undergoing chemotherapy with rituximab containing cytotoxic chemotherapy even if they have never had exposure to lamivudine in the past. In this setting, lamivudine failure due to resistance can develop quickly leading to liver failure that cannot be salvaged with tenofovir. Whether LAM is safe for prophylaxis with rituximab-based cytotoxic chemotherapy for patients with undetectable HBV DNA is unknown, but agents with a high barrier to resistance may be preferable.

## 1. Introduction

Reactivation of hepatitis B infection has been reported in 20%–50% of patients during conventional chemotherapy and up to 80% of patients during rituximab-containing chemotherapy [1]. Chemotherapy-induced reactivation occurs in not only hepatitis B surface antigen- (HBsAg-) positive patients but also in HBsAg-negative/anti-hepatitis B core antibody- (anti-HBc-) positive patients, particularly when rituximab is used [2]. Reactivation is thought to reflect a loss of immune control resulting in abrupt increase in viral replication with or without ALT flare that can be asymptomatic or lead to jaundice in 26%, nonfatal liver failure in 3.7%, and even death in 7% of those with positive HBsAg [3].

The Centers for Disease Control and Prevention (CDC) recommends testing for HBsAg, anti-HBc, and anti-hepatitis B surface Ab (anti-HBs) in persons needing immunosuppressive therapy, including cancer chemotherapy, immunosuppression related to organ transplantation, and immunosuppression for rheumatological, dermatological, or gastroenterological disorders [4].

Many studies have shown the effectiveness of lamivudine to prevent chemotherapy-associated hepatitis B reactivation, with 2 meta-analyses showing a survival benefit to this approach [5, 6]. Katz et al. performed a systematic review and meta-analysis of the effectiveness of lamivudine for all chemoprophylaxis including rituximab-based chemotherapy and found a marked reduction in both clinical and virological reactivations compared to no treatment (OR 0.09; 95% confidence interval (CI) 0.05–0.14 and OR 0.04; 95% CI 0.01–0.20, resp.). All-cause mortality was significantly reduced in the patients who received lamivudine (OR 0.39; 95% CI 0.24–0.62), and prophylaxis also reduced HBV-related mortality (OR 0.20; 95% CI 0.09–0.45) and discontinuation or disruptions of the immunosuppressive treatment [5]. Loomba et al. showed a similar finding that the relative risk for both HBV reactivation and HBV-related hepatitis ranged from 0.00 to 0.21 with preventive lamivudine and none of the patients in the preventive lamivudine group developed HBV-related hepatic failure [6].

The American Association for the Study of Liver Diseases (AASLD) guidelines recommend prophylactic antiviral therapy for HBsAg-positive patients at the onset of immunosuppressive treatment and to continue for 6 months afterwards [7]. Clear guidelines on how to manage patients who are HBsAg negative/anti-HBc positive are lacking, but monitoring is required and it is recommended to start antiviral therapy if HBV DNA becomes detectable (AASLD guidelines Hepatology 2008).

Currently, lamivudine is still used as the first line antiviral agent for prophylaxis to prevent hepatitis B reactivation during immunosuppressive therapy, as it is cheap, safe, and well tolerated. However, the long-term efficacy of lamivudine is limited by the frequent emergence of lamivudine-resistant hepatitis B virus [8]. The incidence of resistance has been reported to be approximately 20% annually in immunocompetent patients receiving long-term treatment [9]. Pelizzari et al. analysed 32 cases of primary lamivudine prophylaxis given to HBV carriers with hematologic malignancies for median followup of 19.5 months and found that the HBV YMMD mutant occurred in only 3.1% of patients with no clinical relevance [10]. Rapid and early emergence of clinically significant resistance is thought to be unlikely during the first year of treatment, particularly for patients with low HBV DNA levels at baseline, and as a result, lamivudine is thought to be a reasonable choice as prophylaxis for most patients during chemotherapy, particularly those scheduled to require prophylaxis for less than one year (AASLD guidelines). This premise is challenged by our recent experience where fatal HBV reactivation was observed despite appropriate lamivudine prophylaxis in two previously treatment-naive individuals undergoing R-CHOP chemotherapy.

## 2. Case Report

### 2.1. Case  1

A 65-year-old Chinese man was diagnosed with stage IIA diffuse, large B-cell lymphoma in April 2011 after presenting with cervical lymphadenopathy. His past medical history was significant for hypertension, thyroidectomy in 2008 for papillary cancer, thalassemia trait, and right inguinal hernia. He was known to have chronic HBV infection but had been told he was an “inactive carrier” and did not receive regular followup and had never received antiviral therapy. He was taking levothyroxine 0.112 mg, valsartan 160 mg, and hydrochlorothiazide 12.5 mg daily. He was also found to have latent TB during screening before R-CHOP chemotherapy. He received the first cycle of R-CHOP on June 2, 2011. He was evaluated by the hepatology service the same week, and lamivudine 100 mg daily was started immediately, on June 10, 2011. Isoniazid 300 mg daily and pyridoxine 25 mg daily were started for latent TB on July 24, 2011, at the recommendation of the infectious disease consultant. Chemotherapy was complicated by febrile neutropenia after the second cycle of R-CHOP, treated with piperacillin-tazobactam (Tazocin) and filgrastim (neupogen), and he subsequently received levofloxacin prophylaxis for the fifth and sixth cycles of chemotherapy. Metformin was also started after the fifth cycle for hyperglycemia. He finished a total of 6 cycles of R-CHOP on September 16, 2011.

Evaluation of the hepatitis B status prechemotherapy showed that he was HBeAg negative and anti-HBe positive with an HBV DNA of 6.65E5 IU/mL and ALT of 43 IU/L. Fibroscan was 4.4 KPa, suggesting F0 (no) liver fibrosis. There was a good initial response to lamivudine, as HBV DNA fell to 2.18E1 IU/mL after 2 months of therapy. His ALT was 36 IU/L after two months, 22 IU/L after three months, and 23 IU/L after 4 months (October 2011) of treatment.

**Figure 1:**
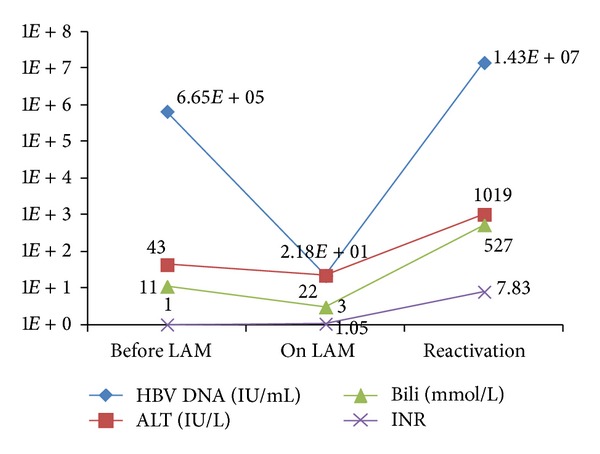
: Biochemical and HBV DNA VL changes in Case  1.

On January 18, 2012, ALT was found to be increased to 79 IU/L on routine bloodwork. Repeated bloodwork on February 2, 2012, 4 months after completing chemotherapy, showed ALT 877 IU/L, AST 1188 IU/L, ALP 118 IU/L, total bilirubin 226 mmol/L, INR 3.21, and HBV DNA 1.34E7 IU/mL (Figure 1). The patient was notably jaundiced but otherwise asymptomatic. He and his family confirmed adherence to lamivudine during and after completing chemotherapy. He was admitted to hospital, despite feeling well, and ALT peaked at ALT 1019 IU/L. INH was also discontinued; however the marked rise in HBV DNA was strongly suggestive of HBV-reactivation-associated hepatitis rather than INH hepatotoxicity. HBeAg remained negative, and genotyping with the INNO-LIPA assay confirmed L80I and M204I mutations, conferring lamivudine resistance. Tenofovir was started on presentation; however there was continued deterioration with investigations showing ALT 578 IU/L, AST 619 IU/L, ALP 121 IU/L, total bilirubin 534 mmol/L, INR 7.05, and HBV DNA 1.94E5 IU/mL. With the continued deterioration despite potent antiviral therapy, a decision was made to add prednisone therapy for a possible anti-inflammatory effect with continuation of tenofovir. Unfortunately there was no clinical response to prednisone, and the patient died on February 17, 2012, from progressive liver failure.

### 2.2. Case  2

A 50-year-old Canadian man of Chinese ancestry was diagnosed with stage III diffuse and large B-cell lymphoma in June 2011 after presenting with cervical lymphadenopathy. His past medical history was significant for gout and psoriasis for which he was using betamethasone/calcipotriol ointment (Dovobet) topically. Hepatitis B was diagnosed during screening for chemotherapy. He received the first cycle of R-CHOP on August 18, 2011, and lamivudine 100 mg daily was started on August 22, 2011. After the first cycle of R-CHOP chemotherapy, he had a significant ALT flare and the second cycle was postponed for 2 weeks with a subsequent 50% dose reduction during the second and third cycles of chemotherapy. He received full dose of chemotherapy after the third cycle. He finished 6 cycles of R-CHOP in February 2012.

Evaluation of the hepatitis B status prechemotherapy showed that he was HBeAg negative. Unfortunately, HBV DNA was not tested, but ALT was 37 IU/L. He did not have clinical evidence of advanced liver fibrosis (platelets 283 × 10E9/mL, AST 21 IU/L, APRI 0.19, suggesting F0 (no) liver fibrosis). After the first cycle of R-CHOP, 24 days after starting lamivudine, his ALT went up to 440 IU/L and AST to 115 IU/L. With the ALT flare, the HBV DNA was measured and found to be 8.90E4 IU/mL. He continued on lamivudine. His ALT subsequently normalized and remained normal during the next 5 cycles of chemotherapy. The HBV DNA was repeated in mid-October 2011 and had declined to 3.96E3 IU/mL, indicating at least a 1.4 log drop with 2 months of lamivudine therapy. The ALT was normal on subsequent testing in January 2012 (30 IU/L) and was found to be slightly elevated at 43 IU/L on March 19, 2012.

**Figure 2:**
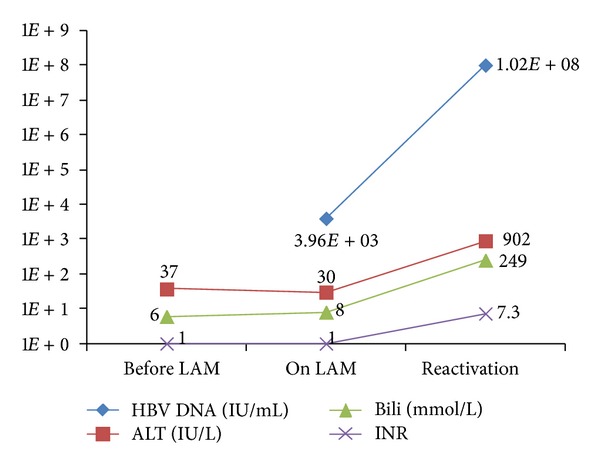
Biochemical and HBV DNA VL changes in Case  2.

At the end of March 2012, he became unwell with fatigue, nausea, and vomiting. On April 10, 2012, two months after completing chemotherapy, while still on lamivudine, he became jaundiced and was admitted to the hospital with acute liver failure. At the time of admission, he had been taking lamivudine for a total of 7 months with confirmed adherence. Investigations revealed a peak ALT of 902 IU/L, AST 612 IU/L, total bilirubin 249 mmol/L, INR 7.3 (Figure 2). HBeAg status was not done, but HBV DNA was found to have risen to 1.02E8 IU/mL. Genotyping with the INNO-LIPA assay confirmed L80I, M80V, and M204V/S mutations, conferring lamivudine resistance. Despite immediate addition of tenofovir, the patient died 3 days later.

## 3. Discussion

Hepatitis B reactivation due to chemotherapy is thought to occur from loss of HBV immune control allowing high-level HBV replication in hepatocytes. The subsequent immune reconstitution after chemotherapy can result in a severe inflammatory syndrome resulting in massive destruction of infected hepatocytes, hepatitis, and liver failure. Loss of HBV-specific immunity seems more likely if rituximab or steroid therapy is combined with standard cytotoxic chemotherapy [11, 12]. Rituximab is a monoclonal anti-CD20 antibody that causes long-lasting B-cell depletion that may last as long as 3 years after stopping the medication [13]. Why rituximab specifically leads to more frequent and potentially more severe HBV-reactivation is unknown but suggests the importance of B cells in HBV immune control.

The two cases presented were very similar (Table 1). Both patients had relatively inactive HBV infection with detectable HBV DNA but normal ALT and no evidence of advanced liver fibrosis. Importantly both patients were treatment-naïve. Both patients reported adherence with medication but neither adhered perfectly to the required monitoring for lamivudine therapy. HBV DNA monitoring is recommended every 3 months during lamivudine treatment. However patient 1 missed his November 2011 visit, which may have identified the presence of genotypic resistance before he presented with clinical liver failure 2 months later. Patient 2 did not have HBV DNA viral load before starting chemotherapy, he missed his March 2012 visit, and he presented with clinical symptoms 1 month later. As has been previously well described, both patients presented with HBV reactivation after completing chemotherapy, presumably due to immune reconstitution leading to hepatitis. Despite taking lamivudine for only 7 months with relatively low viral loads at baseline, both patients developed mutations conferring high-level lamivudine resistance, which ultimately led to HBV reactivation, liver failure, and rapid demise.

Although both patients started lamivudine after starting chemotherapy, the short delay in institution of antiviral therapy was unlikely to have affected their outcome. HBV reactivation typically occurs after the third cycle of rituximab-based chemotherapy [3]. The use of prednisone in the first patient was also somewhat atypical; however this was only added after the patient presented with liver failure and had no response to tenofovir. Antiviral therapy does not work immediately, and the liver dysfunction in the acute setting is due primarily to overwhelming hepatic inflammation. Therefore it was felt that the anti-inflammatory effect of prednisone may be helpful, recognizing that with or without steroids the mortality in this setting was likely to be extremely high.

AASLD guidelines recommend that tenofovir or entecavir could be used as an alternative to lamivudine, particularly in patients who are anticipated to require more than 12 months of therapy in whom there is a higher risk of developing lamivudine resistance. Li et al. compared entecavir and lamivudine in preventing hepatitis B reactivation in lymphoma patients during chemotherapy and found significantly lower rates of hepatitis (5.9 versus 27.0%, *P* = 0.007), hepatitis B reactivation (0 versus 12.4%, *P* = 0.024), and disruption of chemotherapy (5.9 versus 20.2%, *P* = 0.042) in the entecavir-treated patients [14]. There are no specific data comparing tenofovir with other agents in this setting; however its potency, very high barrier to resistance and good safety profile make it a reasonable alternative.

Although only 2 cases, this report highlights that resistance to lamivudine may emerge quickly during immunosuppressive therapy with potentially severe consequences. The use of a more potent agent with a higher barrier to resistance (entecavir or tenofovir) would likely significantly reduce the risk of HBV reactivation due to antiviral resistance. Arguably had both patients adhered strictly to recommended followup, lamivudine resistance may have been recognized before significant hepatitis occurred, which may have led to improved outcomes. However, particularly during cancer chemotherapy with the many potential unforeseen eventualities, followup with scheduled HBV DNA testing may not be strictly followed. Use of an antiviral agent with a lower risk of resistance would reduce the risk that lapses in scheduled followup and would have significant clinical consequences. A trial to compare the efficacy of different antiviral agents to prevent HBV-reactivation during immunosuppressive therapy is unlikely to be performed, and hence inferences may have to be drawn from case reports, case series, and existing data in other clinical settings.

## 4. Conclusion

Lamivudine should not be used for prophylaxis of patients with chronic hepatitis B, especially with detectable HBV DNA, undergoing chemotherapy with rituximab-based chemotherapy even if lamivudine treatment-naive. In this setting, lamivudine resistance may develop quickly with the risk of severe HBV reactivation and subsequent liver failure that cannot be salvaged with tenofovir. If lamivudine is used initially for chemoprophylaxis, close monitoring of HBV DNA is required with consideration of a switch to a more potent agent with a higher genetic barrier to resistance if HBV DNA is not suppressed to undetectable levels within 3 months.

## Figures and Tables

**Table 1:** Basic characteristics and summary of cases.

Characteristics	Case 1	Case 2
Age (years)	65	50
Sex	Male	Male
Ethnicity	Chinese	Chinese
HBV status before chemotherapy	HBeAg negative, Anti-HBe positive, HBV DNA 6.65E5 IU/mL, ALT 43 IU/L	HBeAg negative, Anti-HBe positive, HBV DNA 8.90E4 IU/mL∗, ALT 37 IU/L
Prior hepatitis B treatment	No	No
Clinical evidence of advanced liver fibrosis	Absent	Absent
Fibrosis stage	Fibroscan 4.4 KPa (F0)	APRI 0.19 (F0-1)
Comorbidities	Hypothyroid, hypertension, latent tuberculosis infection	Gout, psoriasis
Medications used	Levothyroxine, valsartan, isoniazid, pyridoxine, metformin	Calcipotriol/betamethasone ointment, Chinese herbal tea
Diffuse, large B-cell lymphoma stage	Stage IIA	Stage III
Lymphoma treatment	R-CHOP x 6	R-CHOP x 6
Lamivudine start date	8 days after first chemotherapy	4 days after first chemotherapy
HBV DNA after 2 months of LAM treatment	2.18E1 IU/mL	3.96E3 IU/mL
Time of HBV reactivation diagnosis	18 weeks after R-CHOP number 6	7 weeks after R-CHOP number 6
Duration of LAM at HBV reactivation	7 months	7 months
Lamivudine resistance pattern	L80I, M204I	L80I, M80V, M204V/S
HBV status at reactivation	HBeAg negative, anti-HBe positive, HBV DNA 1.43E7 IU/mL, ALT 1019 IU/L	HBV DNA 1.02E8 IU/mL, ALT 902 IU/L
Time to death despite tenofovir treatment	14 days	3 days

*HBV DNA at 24 days after starting lamivudine. No baseline HBV DNA was done before lamivudine treatment.
